# Profiling the EU lobby organizations in Banking and Finance

**DOI:** 10.1007/s41109-018-0099-7

**Published:** 2018-10-11

**Authors:** Borut Sluban, Mojca Mikac, Petra Kralj Novak, Stefano Battiston, Igor Mozetič

**Affiliations:** 1 Department of Banking and Finance, University of Zurich, Andreasstrasse 15, Zürich, Switzerland; 2 Department of Knowledge Technologies, Jožef Stefan Institute, Jamova 39, Ljubljana, Slovenia

**Keywords:** Lobby organizations, Banking and finance, Community detection, Co-voting agreement

## Abstract

Creating a map of actors and their leanings is important for policy makers and stakeholders in the European Commission’s ‘Better Regulation Agenda’. We explore publicly available information about the European lobby organizations from the Transparency Register, and from the open public consultations in the area of Banking and Finance. We consider three complementary types of information about lobbying organizations: (i) their formal categorization in the Transparency Register, (ii) their responses to the public consultations, and (iii) their self-declared goals and activities. We consider responses to the consultations as the most relevant indicator of the actual leaning of an individual lobbyist.

We partition and cluster the organizations according to their demonstrated interests and the similarities among their responses. Thus each lobby organization is assigned a profile which shows its prevailing interest in consultations’ topics, similar organizations in interests and responses, and a prototypical question and answer. We combine methods from network analysis, clustering, and text mining to obtain these profiles. Due to the non-homogeneous consultations, we find that it is crucial to first construct a response network based on interests in consultations topics, and only then proceed with more detailed analysis of the actual answers to consultations. The results provide a first step in the understanding of how lobby organizations engage in the policy making process.

## Introduction

Policy changes and initiatives are often triggered by the stakeholders that are going to be affected by those future policies, e.g. a specific sector of the industry. In democratic countries policy makers typically consult a limited number of experts and the largest stakeholders directly involved before issuing a new policy proposal. However, this process leaves citizens and smaller stakeholders underrepresented in the process of policy-shaping. Therefore in many countries, governments are working on improving the communication with citizens and stakeholders to increase their involvement in the law-making process. As an example, the European Commission (EC) has been making a significant effort to engage an increasing number of citizens in the EU law-making process by means of open public consultations (European Political Strategy Centre 2018). This was formerly known as the “Your Voice in Europe” initiative where citizens and stakeholders were encouraged to provide feedback to policy proposals by means of responses to the open public consultations. Typically, the responses are limited to a few hundred, mostly coming from the lobbying organizations that are active in the specific policy areas.

There are several empirical studies on interest group mobilization in the EU examining the number and type of interest groups politically active in the EU. The EC lobbying register was inspected in (Coen and Katsaitis 2013) to assess the density and diversity of the interest group population per policy domain. The density of interest organizations per economic sector in the EU is explained in (Berkhout et al. 2015) on the basis of political and economic institutional factors. Based on an analysis of the EC online consultations, in (Rasmussen et al. 2014) it was found that organized interests can potentially act as a transmission belt between the public and the decision makers. Higher mobilization rates were found on those issues that fall within policy areas that are regarded as salient by the general public and those with consequences for budgetary spending. However, little research has been carried out so far on the structure of networks in which lobbies operate (Wolf et al. 2014). In (Zeng and Battiston 2016) data from the EU Transparency Register (TR) and the Orbis database was combined to construct a multiplex lobby network consisting of the affiliation, shareholding, interlocking and client relations between lobby organizations. No simple relation was found between the network centrality of the organizations and their size, for instance in terms of funds deployed in lobbying. However, each network layer was found to provide complementary information to characterize the influence of the organizations. Regarding inter-layer influence, a comprehensive review of multilayer networks can be found in (Kivelä et al. 2014). Other related previous works looked at the community structure of networks of corporations arising from ownership relations (Vitali and Battiston 2014) and interlocking directors (i.e., common members in board of directors, (Piccardi et al. 2010; Heemskerk and Takes 2016)). However, to the best of our knowledge, the authors did not consider the links arising specifically among lobbying organizations in the context of their activity in the policy making process. They have all used variants of the Louvain algorithm (Blondel et al. 2008) to detect the communities. A method to generate statistically validated networks as a projection of a bipartite graph is given in (Tumminello et al. 2011), and can be applied to bipartite lobbyist-policy, lobbyist-consultation or lobbyist-position networks.

In our closely related work (Sluban et al. 2017) we already analyzed how lobby organizations respond to the EC’s public consultations in the area of Banking and Finance. We considered 363 lobby organizations from the Transparency Register, their responses to 12 consultations, their formal categorization into organization types, and their self-described areas of interest and activities. We constructed a network of organizations which showed similarities between their policy positions raised in the consultation. We compared the communities of the preference patterns network with predefined organization types and organization clusters calculated from their textual descriptions. We found relatively low values of the comparison measures, and concluded that the declared goals and activities do not align well with the preference patterns as demonstrated in responses to consultations. This motivated the current study where we re-focus our research on profiling the lobby organizations with respect to their responses to consultations.

In this study we extend the set of consultations to 21 and the number of lobby organizations to 565. We also shift the focus of the analysis. Previously we focused on the themes of consultations in which the organizations participated by comparing three data sources (categorizations, self-descriptions and responses to consultations) pairwise. In this paper we focus on the analysis of profiles of the lobby organizations themselves. As we concluded in our previous work, we consider responses to consultations the most relevant indicator of the actual leanings of individual lobby organizations. We refine our profiling method by focusing on the answers to the consultations. Information whether an organization participated in a particular consultation is, of course, interesting, however, analyzing the actual answers sheds more light on the viewpoint of a certain organization with regard to the questions. Thus, the profiles of lobby organizations are characterized by the clusters of organizations with similar interests and actual responses (co-voting) to consultations. Additionally, we characterize each co-voting cluster with prototypical organization and with questions/answers with the highest agreement in a cluster. As in our previous work, we re-analyze their self-described areas of interest and activities in order to get yet another view on the organizations.

The paper is structured as follows. In “Data and preprocessing” section we provide details about data sources, in particular the Transparency Register and the 21 public consultations. “Profiling lobby organizations” section describes main methods used and the results. In “Topic communities of responding organizations” subsection we create a response network between the organizations, and detect communities with similar interests. “Clusters of co-voting organizations” subsection describes how to further partition the communities into clusters of organizations with similar answers to the same consultation questions. In “Characterizing clusters by typical organizations and questions/answers” subsection we additionally characterize clusters by their medoid organizations and most typical questions and answers. In “Clustering of descriptions” subsection we show how to process textual data to create tag clouds of the similar lobby organizations according to their self-descriptions. “Interactive exploration of the lobby profiles” section gives an overview of and a link to the *Lobby Profile Explorer*. This is an openly accessible web application which supports interactive exploration of lobby organizations and their responses to public consultations. We conclude the paper in “Conclusions” section with lessons learned.

## Data and preprocessing

We focus on lobby organizations registered in the EU Transparency Register (2018) and active in the area of Banking and Finance (Consultations (banking and finance) 2018). We analyze and compare three aspects of these organizations:

- their formal categorization,
- their responses to public consultations and
- their self-described goals and activities.

The study covers 565 organizations that responded to multiple choice questions in 21 public consultations, from June 2014 to November 2017.

**The transparency register** was set up by the European Parliament and the EC to increase open access to information about “what interests are being pursued, by whom and with what budgets”. The Transparency Register provides information about a main category and subcategory in which an organization is registered (Transparency Register Data 2018). Distribution of the organizations over the categories and subcategories is shown in Table 1. The majority (74%) of the 565 organizations are in the “II - In-house lobbyists and trade/business/professional associations” main category. Therefore, in subsequent analyzes, these organizations are further categorized in more specific subcategories of “II”.

**Table 1:** Transparency Register categories and subcategories, and the distribution of the 565 lobby organizations (Org) analyzed in this study

Category/subcategory		Abbreviation	Org	Share
I -	Professional consultancies/law firms/self-employed	Consultant	8	1%
	consultants			
II -	In-house lobbyists and trade/business/professional		417	
	associations			
	- Companies and groups	Company	146	26%
	- Other organisations	Other lobbyist	17	3%
	- Trade and business associations	Association	204	36%
	- Trade unions and professional associations	Trade union	50	9%
III -	Non-governmental organisations	NGO	93	17%
IV -	Think tanks, research and academic institutions	Think tank	19	3%
V -	Organisations representing churches and religious		0	
	communities			
VI -	Organisations representing local, regional and municipal	Public auth.	28	5%
	authorities, other public or mixed entities, etc.			
Total			565	100%

We consider main categories of the organizations, except for the largest category II, where we consider its subcategories (column Abbreviation)

**Public consultations** (Public Consultations 2018) are used by the EC to involve citizens and stakeholders in the law-making process. From June 2014 to November 2017, there were 21 relevant consultations in the area of Banking and Finance. The list of analyzed consultations is shown in Table 2. On average, there are 44 questions per consultation, but the number varies from 3 to 151. There are typically 3 or 4 possible answers to a question. The actual number of questions and possible answers per consultation are also in Table 2.

**Table 2:** Public consultations analyzed in this study and the number of lobby organizations (Org) which responded to them

Consultation		Org	Quest	Ans
#1	Consultation on the Equivalence of third country regimes	10	4	8
	regarding the country by country reporting by extractive and			
	forestry industries			
#2	Consultation on the Review of the Prospectus Directive	77	51	123
#3	Public consultation Building a Capital Markets Union	190	6	12
#4	Public consultation on further corporate tax transparency	81	24	96
#5	Public consultation on long term finance	40	17	37
#6	Public consultation on covered bonds in the European Union	36	82	167
#7	Public consultation on the review of the European Venture	21	13	26
	Capital Funds (EuVECA) and European Social			
	Entrepreneurship Funds (EuSEF) regulations			
#8	Green Paper on retail financial services	112	32	123
#9	Public consultation on non-financial reporting guidelines	134	34	201
#10	Public consultation on cross-borders distribution of	32	44	88
	investment funds			
#11	Public consultation on a potential EU personal pension	7	79	325
	framework - consumer organisations			
#12	Public consultation on a potential EU personal pension	47	51	200
	framework - stakeholders			
#13	Review of the EU Macro-prudential framework	33	30	120
#14	Mid-term evaluation of the Connecting Europe Facility (CEF)	34	36	155
	- general questionnaire			
#15	Mid-term evaluation of the Connecting Europe Facility (CEF)	47	146	612
	- technical questionnaire			
#16	Capital Markets Union mid-term review 2017	117	5	10
#17	Whistleblower protection	53	151	608
#18	Operations of the European Supervisory Authorities	104	3	6
#19	FinTech a more competitive and innovative European	96	23	46
	financial sector			
#20	Development of secondary markets	27	11	22
#21	Post-trade in a Capital Market Union dismantling barriers	39	86	310
	and strategy for the future			
Total		1,337	928	3,295

For each consultation we also provide the number of questions (Quest) and the total number of possible answers (Ans) to them

We extracted the data from the consultation questionnaires for organizations which provided at least one answer to a multiple choice question. This allows us to find exact matches of their responses in contrast to open ended questions where comparison of two answers is more involved. Each response to a consultation is transformed into a binary vector denoting which of the answers to the multiple choice questions are provided. For each organization participating in at least one consultation, a joint vector from all 21 consultations is created. We omit the non-informative answers, such as “No Answer”, “Don’t know”, “No opinion”, “Not relevant”, etc. The result is a 3,295-dimensional binary vector for each organization called *voting vector*. The voting vector is subsequently used to compare similarities and differences in answers between different organizations that have similar interests. In particular, the analysis consists of two steps. First we identify organizations with similar interests, i.e., those that responded to the same consultations. We construct a response network and compute topic communities, combining organizations with similar interests. Then we analyze each topic community separately, by comparing the voting vectors of member organizations. We thus combine two views on the consultations: (i) interest in the topic, where we ignore the complexity of questions and answers, and (ii) actual answers, where voting vectors are compared.

**Goals and activities.** During the registration in the Transparency Register, an organization itself describes its goals and main activities. We extract all these descriptions and merge them into a single text document for each organization. We remove any URLs as we consider only the content, and do not inspect links to other sources. We take into account only English documents. Each document is split into sentences; this is necessary since some documents contain text written in more than one language. Eventually, we consider only organizations which have English descriptions that are longer than 50 characters. As a consequence, from the initial 618 organizations which responded to consultations we eliminated 53, thus considering 565 organizations in further analyzes. The language detection and text processing is implemented in the LATINO text mining library (2018).

## Profiling lobby organizations

This section presents methods applied and the main results. In “Topic communities of responding organizations” subsection we start from the list of consultations and organizations which responded to them. We create a *response network* which links organizations responding to the same consultations, and detect communities in it. A community corresponds to a set of organizations which are interested in consultations about similar topics, and are therefore named *topic communities*. In “Clusters of co-voting organizations” subsection we further refine the analysis, and inspect the actual answers to the consultation questions. Based on the similarity of answers, each topic community is partitioned into clusters, named *co-voting clusters*. Note that the size and complexity of an individual consultation is irrelevant to detect topic communities, but it is crucial when computing co-voting clusters within topic communities. Thus, both aspects are taken into consideration in a balanced way: interests in consultations and topics, and the actual answers via voting. Each co-voting cluster is additionally characterized in “Characterizing clusters by typical organizations and questions/answers” subsection by its medoid organization, and a question and answer most agreed upon. Finally, we apply text mining to self-descriptions of the lobby organizations, cluster them and produce descriptive *tag clouds* (“Clustering of descriptions” subsection).

### Topic communities of responding organizations

The goal of this subsection is to group lobby organizations into communities with similar interests with regard to the consultations. We start with a bipartite graph comprised of 565 organizations and 21 consultations, where there is an edge if an organization responded to a consultation. The majority of organizations (51%) responded to one consultation only, 16% responded to two consultations, 8.5% to three, 5.3% to four, and so forth. From Table 2 we can see that consultations with the highest response rate are consultations #3, #9, #16 and #8. As our goal is to profile and describe activities of the organizations, we project the bipartite graph to a weighted response network. Nodes in the network (*N*=565) represent the organizations, and edges (*M*=90,954) reflect their participation in the same consultation. Two nodes are linked by an edge if the organizations responded to the same consultation where the weight is the number of the same consultations.

The network is constructed and analyzed using Gephi (Bastian et al. 2009). The response network has a density score of 0.285. The degree distribution is as follows: share of nodes with a degree ≤ 100 is 32.6%, between 100 and 200 is 35.2%, between 200 and 300 is 20.5%, between 300 and 400 is 10.1%, and there are 9 nodes with a degree > 400 (1.6%). The highest node degree is 451, and the average weighted node degree is 115.4. The response network has a clustering coefficient of 0.867, relatively high.

In the response network, we identify communities of organizations which exhibit similar interests, i.e., they respond to the same consultations. We detect the communities by applying the Louvain method (Blondel et al. 2008; Lambiotte et al. 2009). The method was applied several times with different parameter values, and eventually the default parameters, resulting in maximum modularity, were used: randomize=On, use edge weights=On, resolution=1.0. The community detection yields five non-overlapping communities with the modularity value of 0.227. The Louvain method is non-deterministic and running it multiple times results in slightly different community partitions. We check the robustness of the results by applying the method 50 times with random seed and the same parameters. The similarity of the 50 resulting partitions is then compared by the Rand index (Rand 1971). It has a value between 0 and 1, with 0 indicating that the two community partitions are totally different and 1 indicating that two partitions are the same. We calculate the Rand index pairwise and get relatively high values (average Rand index = 0.892 with 95% CI = [0.89, 0.90] and p-value < 2.2·10^−16^). This indicates that the partitioning in the five detected communities is relatively stable.

**Fig. 1:**
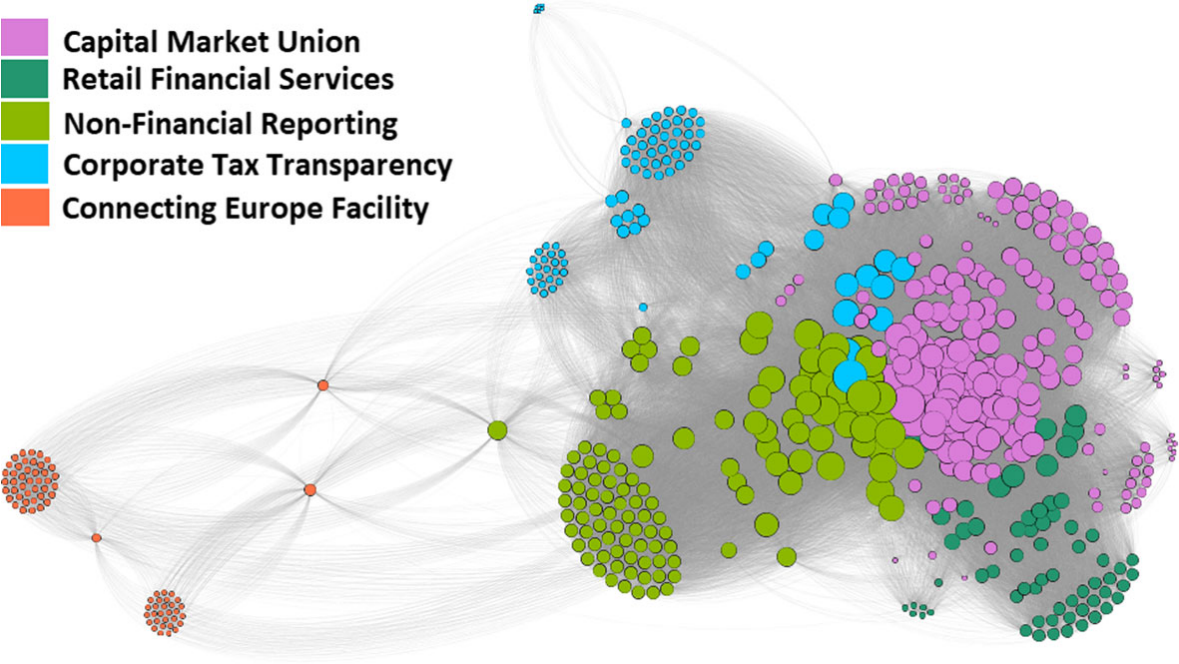
Response network of the 565 lobby organizations. Two organizations are linked if they respond to the same consultation. Different colors denote the five detected communities. The communities are labeled by the prevailing topics in common consultations. Node size is proportional to the number of consultations to which the organization responded

The response network with the detected communities is depicted in Fig. 1. Different node colors correspond to the five detected communities. The network visualization is produced using the ForceAtlas2 layout in Gephi.

**Fig. 2:**
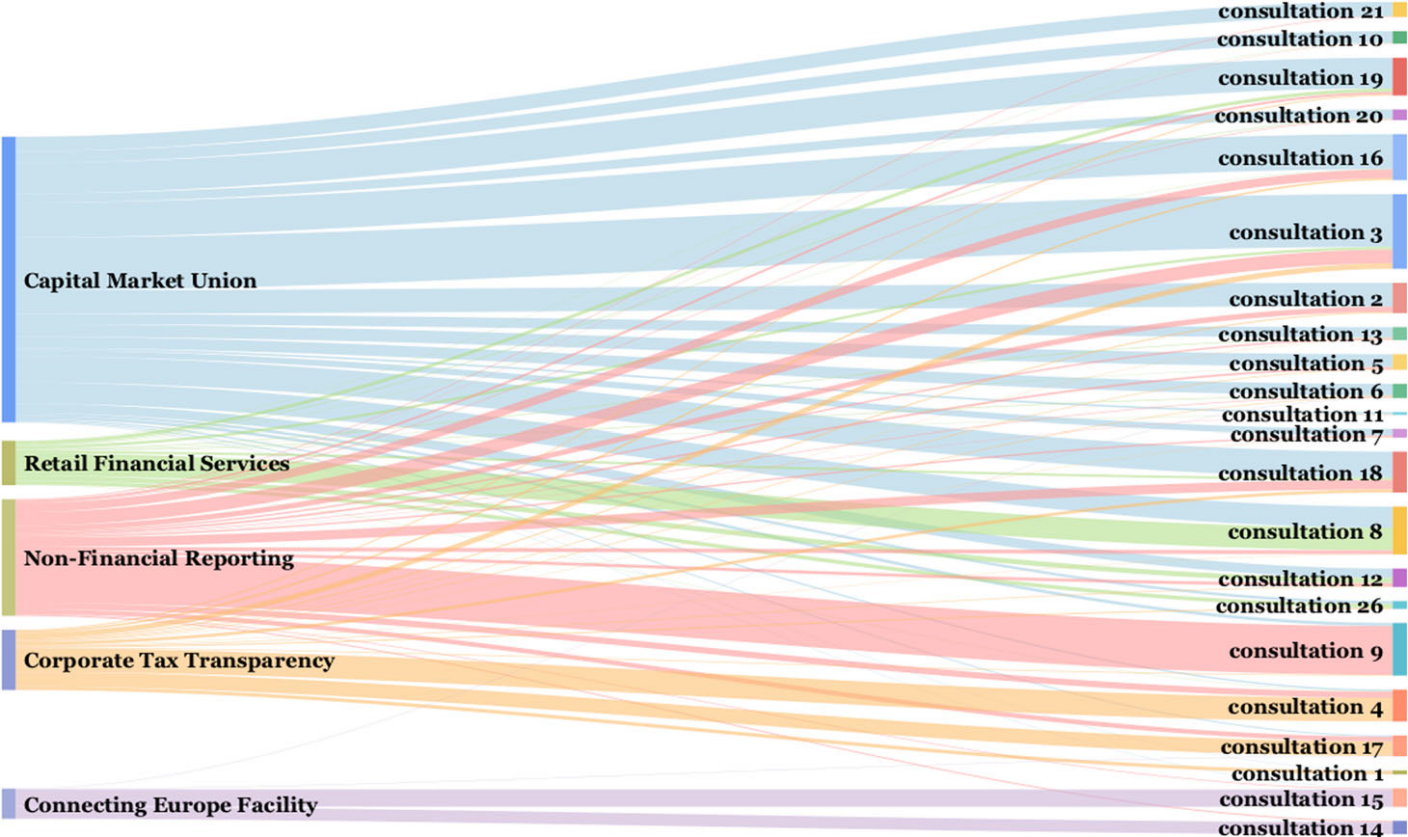
Relations between the topic communities and consultations. The Sankey diagram links detected topic communities (left-hand side) to the consultations (right-hand side). The thickness of a link corresponds to the number of organizations that responded to a consultation. The diagram clearly shows the overlaps between the topic communities

The detected communities partition the set of lobby organizations into five non-overlapping sets. Each community represents participation in common consultations and engagement in certain topics. However, organizations also respond to some consultations outside their core community, therefore the correspondence between the communities and consultations is not one-to-one. We argue that computing the non-overlapping communities in the first phase, and then showing explicit overlaps across consultations provides better insight than detection of overlapping communities. In Fig. 2 we show for each community the distribution of its members’ responses to the individual consultations. The communities are labeled according to their main topics of engagement, therefore they are called *topic communities*. The correspondence between the communities and consultations can be intuitively presented with the Sankey diagram (Sankey Diagram 2018). The proportional flow diagram shows how many organizations from different communities responded to individual consultations.

We observe that the organizations comprising the first and the largest community are mainly focused on two consultations: #3 (*Public consultation Building a Capital Markets Union*) and #16 (*Capital Markets Union mid-term review 2017*). We labelled this as the *Capital Market Union* community. In the second largest community, the main topic of interest is consultation #9 (*Public consultation on non-financial reporting guidelines*), therefore we labelled this community as *Non-Financial Reporting*, and so forth. Detailed results for all the communities are in Table 3 where we also show the top consultations to which a large number of organizations responded. We can observe that communities form around consultations that are represented by higher degree nodes (#3, #9, #16, #8) in the bipartite graph.

**Table 3:** Detected topic communities of the lobby organizations, their number and share in each community, and top consultations which received the most responses (the number of responding organizations is in parentheses)

Community	Org	share	Top consultations (Org)
1. Capital market union	213	38%	#3 (134), #16 (89), #19 (78), #18 (66)
2. Non-financial reporting	124	22%	#9 (124), #3 (35)
3. Corporate tax transparency	91	16%	#4 (60), #17 (37)
4. Retail financial services	64	13%	#8 (57)
5. Connecting Europe facility	73	11%	#15 (44), #14 (31)
Total	565	100%	

### Clusters of co-voting organizations

Topic communities are groups of lobby organizations which respond to common consultations. In this subsection we analyze their actual answers to questions in the consultations. We use the high-dimensional *voting vectors* to compute co-voting similarities between organizations. Within each topic community we form clusters of organizations with similar responses to consultations, i.e., similar voting vectors.

Let **a** and **b** denote voting vectors of organizations A and B, respectively. We define co-voting similarity between A and B as the cosine similarity between vectors **a** and **b**:

$$\cos (\angle(\mathbf{a},\mathbf{b})) = \frac{\mathbf{a}\cdot \mathbf{b}}{|\mathbf{a}|\cdot|\mathbf{b}|}. $$

Cosine similarity is calculated as the normalized dot product of **a** and **b**. It ranges between 0 and 1, where 0 indicates complete dissimilarity, and 1 complete agreement. For clustering, we define the distance between two voting vectors as:

$$\text{distance}(\mathbf{a}, \mathbf{b}) = 1 - \cos (\angle(\mathbf{a},\mathbf{b})). $$

We apply Ward’s method (Ward 1963) with agglomerative hierarchical clustering over the voting vectors. Ward’s method, precisely called minimum variance method, minimizes the total within-cluster variance. The resulting hierarchy of clusters can be represented by a dendrogram, where any level of agglomeration can be selected. We decided to uniformly split each topic community into three co-voting clusters. It turns out that after partitioning into three clusters, at least one cluster has considerably higher co-voting agreement than the original community. Clusters could be further partitioned, but one should avoid too small clusters. The resulting clusters are shown in Table 4.

**Table 4:** Clusters within topic communities

Cluster	Org	*A* *l* *p* *h* *a*	*J* *S* *D*	Top TR category share	
1. Capital market union	213	0.175			
1.1	42	0.227	0.117	Association	64%
1.2	18	0.624	0.071	Company	39%
1.3	153	0.143	0.025	Association	39%
2. Non-financial reporting	124	0.647			
2.1	70	0.584	0.070	NGO	34%
2.2	48	0.682	0.052	Association	48%
2.3	6	0.492	0.550	Trade union	83%
3. Corporate tax transparency	91	0.201			
3.1	38	0.253	0.074	Association	55%
3.2	20	0.563	0.320	NGO	55%
3.3	33	0.424	0.229	Trade union	45%
4. Retail financial services	64	0.114			
4.1	43	0.115	0.101	Association	63%
4.2	9	0.390	0.523	NGO	55%
4.3	12	0.554	0.225	Association	42%
5. Connecting Europe facility	73	0.181			
5.1	29	0.313	0.220	Public auth.	38%
5.2	15	0.168	0.184	Company	60%
5.3	29	0.247	0.096	NGO	38%

Each community is partitioned into three clusters w.r.t. the co-voting agreement. For each cluster there is the number of organizations in the cluster (Org), the co-voting agreement in terms of Krippendorff’s *Alpha*, and the Jensen-Shannon divergence (*JSD*) to the overall distribution in terms of the TR categories. The last two columns show the dominant TR category in the cluster, and its share of the organizations in this category

The level of selected agglomeration is validated by a network analysis. We construct yet another network with organizations as nodes and values of cosine similarity as weights on the edges. We apply the Louvain method on each individual community. The *Capital Market Union* community is partitioned in seven subcommunities with modularity of 0.181, the *Non-Financial Reporting*, *Corporate Tax Transparency*, and *Retail Financial Services* community are partitioned into three subcommunities with modularity levels of 0.114, 0.316, and 0.189, respectively. *Connecting Europe Facility* is partitioned in two subcommunities with modularity of 0.335. We compared each community partitioning to the co-voting clusters by Rand index. The values of Rand index are 0.587, 0.860, 0.737, 0.802, 0.803, respectively, relatively high for all the communities except for the first and the largest *Capital Market Union* community. We can conclude that a uniform agglomeration into three co-voting clusters is a sensible choice for all the communities, except for the first community. However, for the sake of uniformity and to avoid too many clusters with a small number of members in each, we settled for three co-voting clusters also in this case. This is not an optimal choice and in the future a better criterion to select an appropriate number of co-voting clusters should be devised.

We analyze several properties of the co-voting clusters: level of agreement between the organizations, distribution of the Transparency Register categories, and the dominant category in each cluster.

The co-voting agreement between organizations in a co-voting cluster is computed by Krippendorff’s *Alpha* agreement measure (Krippendorff 2013). *Alpha* is typically used as a measure to quantify the extent of agreement among human raters. When raters agree perfectly, *Alpha*=1, and when the level of agreement equals the agreement by chance, *Alpha*=0. Besides its typical applications, *Alpha* was already used to quantify the agreement between annotators in machine learning (Mozetič et al. 2016), and co-voting agreements and disagreements in the European Parliament (Cherepnalkoski et al. 2016). In our case, *Alpha* measures the level of agreement between answers to consultations (see Table 4).

In general, we observe fairly low values of *Alpha*, in comparison to other domains. In the case of public consultations, the questionnaires are thematically very broad, and we are applying the Krippendorff’s *Alpha* to non-typical data. In some clusters, the degree of agreement remains at the level of their respective communities, while in others the agreement increases. In particular, in clusters 1.2, 3.2, and 4.3 *Alpha* considerably increases as a community is partitioned into co-voting clusters. In certain topic communities (e.g., *Non-Financial Reporting*) the agreement is already high, and there is no significant difference between the clusters and the overall community agreement. We can infer that such topics are sufficiently noncontroversial, and that the responding organizations have a common view on the subject.

Another interesting property of the co-voting clusters is the distribution of the Transparency Register (TR) categories within them. The last two columns in Table 4 show the prevailing TR category and its share in each cluster. We also compare the distribution of the TR categories within each cluster to their overall distribution in TR. We measure the similarity between the two distributions (*P, Q*) by Jensen-Shannon divergence (*JSD*) (Lin 1991):

$$\mathit{JSD}(P,Q) = H\left(\frac{1}{2} P + \frac{1}{2} Q\right) - \frac{1}{2}(H(P) + H(Q)) $$

where *H*(*P*) is the Shannon entropy of a discrete distribution *P*. *JSD* ranges between 0 and 1, where 0 indicates identical distributions, and 1 completely different distributions. We note that some clusters, e.g., 2.3 and 4.2, have very different distribution of the TR categories in comparison to the prior.

### Characterizing clusters by typical organizations and questions/answers

In this subsection we additionally characterize the co-voting clusters. Table 5 shows representative organizations for each cluster. Technically, an organization is a medoid of a cluster if it has minimal average co-voting distance to all other organizations in the cluster. Note that medoids do not always belong to the dominant TR category in the cluster (see Table 4).

**Table 5:** The medoid organizations of each co-voting cluster

Cluster	Organization and country		TR category
1.1	Die Deutsche Kreditwirtschaft	DE	Association
1.2	Association Luxembourgeoise des Fonds d’Investissement	LU	Association
1.3	London Stock Exchange Group	UK	Company
2.1	Allianz SE	DE	Company
2.2	Deutsches Aktieninstitut	DE	Association
2.3	Vereinte Dienstleistungsgewerkschaft	DE	Trade union
3.1	Confederation of Danish Industry	DK	Association
3.2	BEPS Monitoring Group	UK	NGO
3.3	Transparency International	BE	NGO
4.1	PayPal Se Belgian Branch	LU	Company
4.2	TGTEuropeORG	DE	Public auth.
4.3	Association of International Life Offices	BE	Association
5.1	STRING	DK	Public auth.
5.2	OGP Gaz-System S.A.	PL	Company
5.3	ZERO - Associaçao Sistema Terrestre Sustentável	PT	NGO

Another interesting characterization are the questions and answers with the highest agreements per each cluster. When the majority (at least 75%) of the organizations in the cluster responded to a consultation, we extracted the question/answer that was the most unanimous. The results are given in Table 6.

**Table 6:** Prototypical questions and answers for each co-voting cluster

Cluster	Consultation/QuestionID: Question text	Answer
1.1	#8/Q7: Is the quality of enforcement of EU retail financial services legislation across the EU a problem for consumer trust and market integration?	Yes
1.2	#12/Q10: What information, in your opinion, is most relevant to individual savers before signing up to a product?	The tax regime for contributions, returns and pay-outs (very important)
1.3	#3/Q23: Are there mechanisms to improve the functioning and efficiency of markets not covered in this paper, particularly in the areas of equity and bond market functioning and liquidity?	Yes
2.1	#9/Q1: What aspects of disclosure of non-financial information do you think that should be addressed by the guidelines?	Materiality/Relevance
2.2	#9/Q8: How do you think that the guidelines should relate to existing national, international or other EU-based frameworks (such as UN Global Compact, the UN Guiding Principles on Business and Human Rights, OECD guidelines for multinational enterprises, the ILO Tripartite Declaration of principles concerning multinational enterprises and social policy, EMAS, etc.)?	The guidelines should make reference to other frameworks where addressing concrete matters or specific issues
2.3	#9/Q3: In your opinion, what features make a piece of information relevant (or material) for the purposes of the non-financial statement?	Necessary to understand how the company manages non-financial risks
3.1	#4/Q17: Is there a risk that tax transparency towards the public could have other unintended negative consequences on companies?	Yes
3.2	#4/Q2A: Do you agree with the following objectives: To increase pressure on enterprises to geographically align taxes paid in a country with actual profits, by enhanced scrutiny and decisions of either citizens or tax authorities (enterprises should pay tax where they actually make profit)?	Yes
3.3	#17/Q: Do you think that whistleblowing should be protected?	Yes
4.1	#8/Q2A: What are the barriers which prevent firms from directly providing financial services cross-border?	Language, Differences in national legislation, Additional requirements imposed by national regulators
4.2	#8/Q6: Do customers have access to safe, simple and understandable financial products throughout the European Union?	No
4.3	#12/Q11: What information, in your opinion, is most relevant to individual savers during the lifetime of the product?	Level of protection provided (very important)
5.1	#15/Q2: In your opinion, how important is each of the following CEF objectives to the goal of developing trans-European transport, energy and telecommunications networks?	Develop the physical transportation, energy and telecommunications infrastructure (very important)
5.2	#15/Q1: In your opinion, is there still a need to continue financial support from the EU budget for the development of trans-European networks?	Yes
5.3	#14/Q1: In your opinion, should investing in the fields of transport, energy and telecommunications be an EU priority?	Yes

Selected are questions from individual consultations, where the answers show the highest level of co-voting agreement between the organizations in the cluster

We can draw some overall conclusions about the five topic communities and their further refinements into the co-voting clusters.

The largest community, *Capital Market Union*, is composed of a wide, not clearly differentiated interests, comprised of various associations and companies. Organizations in the *Capital Market Union* community, are relatively active — on average they responded to 3.4 consultations. The level of agreement is low in this community, except in cluster 1.2 where a somewhat higher agreement can be attributed to the cluster’s small size (only 18 members). As already noted, the partitioning of this community into three co-voting clusters is not optimal, and the cluster 1.3 should probably be further partitioned into sub-clusters.

The second community, *Non-Financial Reporting*, is homogeneous with a high degree of agreement between its member organizations. All organizations in this community participated in consultation #9 (*Public consultation on non-financial reporting guidelines*). Most of the organizations in a co-voting cluster 2.1 are of opinion that the most important non-financial aspect of disclosure should be relevance/materiality. In this cluster, organizations participated on average in 2.2 consultations. The cluster is mainly comprised of associations. Cluster 2.2, mainly comprised of companies, participated in 2.6 consultations on average. Cluster 2.3 is very small (6 organizations only, mainly trade unions) who agree that companies should have better understanding of the non-financial risks.

The third community, *Corporate Tax Transparency*, is the most interesting one. Two of its clusters (3.1 and 3.2) comprise organizations with almost directly opposing responses to consultations. In cluster 3.1 we observe opposition to the tax transparency, whereas cluster 3.2 argues for responsible taxation wherever enterprises make profit. The specific question that highlights these differences is whether there is a risk that tax transparency towards the public carries unintended negative consequences. The majority of lobbyists in cluster 3.1 are associations, and their answer is unanimously positive, i.e., tax transparency may have unintended consequences. The majority in cluster 3.2 are NGOs, and they answer the same question negatively. The third cluster 3.3 is not as distinctive.

Organizations that form two of the smallest communities, *Retail Financial Services* and *Connecting Europe Facility*, have very specific profiles, with narrowly expressed interests.

The *Retail Financial Services* community is comprised of 64 organizations. In this community, organizations participated in only 1.7 consultations on average, most of them (89%) participated in consultation #8 (*Green Paper on retail financial services*), their overall agreement is relatively low. Organizations in the co-voting cluster 4.1, mainly comprised of associations, are of opinion that the main barriers preventing firms from providing cross-border financial services are language, differences in national legislation, and additional requirements imposed by national regulators. Organizations in the co-voting cluster 4.2, mostly NGOs, believe that customers don’t have access to safe, simple and understandable financial product throughout EU. All companies in the co-voting cluster 4.3, with a relatively high agreement level, participated also in consultation #12 (*Public consultation on a potential EU personal pension framework-stakeholders*). They agree that the level of protection during the lifetime of a product is most relevant to individual savers.

In the *Connecting Europe Facility* community all organizations, but three, participated in one consultation only. The two co-voting clusters 5.1 and 5.2 in consultation #15 (*Mid-term evaluation of the Connecting Europe Facility (CEF) - technical questionnaire*), and the co-voting cluster 5.3 in consultation #14 (*Mid-term evaluation of the Connecting Europe Facility (CEF) - general questionnaire*). This is a very narrow and specific theme which seems to be of no interest to a wider range of organizations. The level of agreement is low in every co-voting cluster, but the members mostly agree on the following. In cluster 5.1, surprisingly comprised mainly of public authorities, the organizations are engaged in developing the physical transportation, energy and telecommunications infrastructure. In cluster 5.2 the organizations are of opinion that there is still a need to continue financial support from the EU budget for developing trans-European networks. Organizations in cluster 5.3 believe that investing in the fields of transport, energy and telecommunications should be the EU priority.

From this analysis, it emerges how the co-voting patterns across communities are heterogeneous. In some cases, as for the third community, *Corporate Tax Transparency*, there is a clear difference in voting between groups identified ex-ante based on their TR category (i.e., NGO’s versus business associations). In other cases, as for the second community, *Non-Financial Reporting*, the same ex-ante categories do not display significantly different co-voting behaviour. This heterogeneity can be explained in part by the level of controversy of the consultation topics. For instance, the topic of tax transparency is known to create opposing views between civic society and corporate lobbyists. In contrast, the topic of corporate social responsibility is known to find support of many stakeholders of the corporate sector because the idea that firms should disclosure non-financial information, relevant to social and environmental aspects and sustainability, is perceived as an opportunity for building reputation among consumers and customers. However, the level of controversy is not fully known ex-ante by the policy makers. Therefore, consultations provide a useful indication to policy makers on which points exactly the controversies arise. On the other hand, the heterogeneity of patterns can also be explained by the fact that both NGO’s and corporations have different purposes and strategies in the policy making process which cannot be simply classified ex-ante.

### Clustering of descriptions

The goal of this subsection is to get yet another view on the properties of the analyzed lobby organizations. We apply text mining tools to extract typical features from descriptions of goal and activities, that the organizations themselves provided in the Transparency Register. In particular, we apply the *K*-means clustering (Hartigan 1975) that partitions all the provided descriptions into *K* clusters. Organizations with similar goals and activities are then grouped in the same cluster.

First, textual descriptions are preprocessed by standard text preprocessing (Feldman and Sanger 2006) methods. For each description (only parts in English are considered), the text is tokenized and stemmed, stop words are removed, unigrams and bigrams are formed, and feature vectors are constructed by the TF-IDF weighting scheme and normalization. The resulting bag-of-words vectors are an input to the *K*-means clustering algorithm. We then apply the *KMeansClusteringFast* algorithm from the LATINO library (LATINO text mining library 2018). The value of *K* is set to 5.

We tested different values of *K* in the range between 2 and 10, with ten different seeds for an initial clustering setup. The quality of the resulting clusters was estimated by the Silhouette coefficient (Rousseeuw 1987). Since there was no significant difference between the quality of clusters for *K* between 2 and 6, we selected *K*=5 to match the number of detected topic communities (see “Topic communities of responding organizations” subsection).

**Fig. 3:**
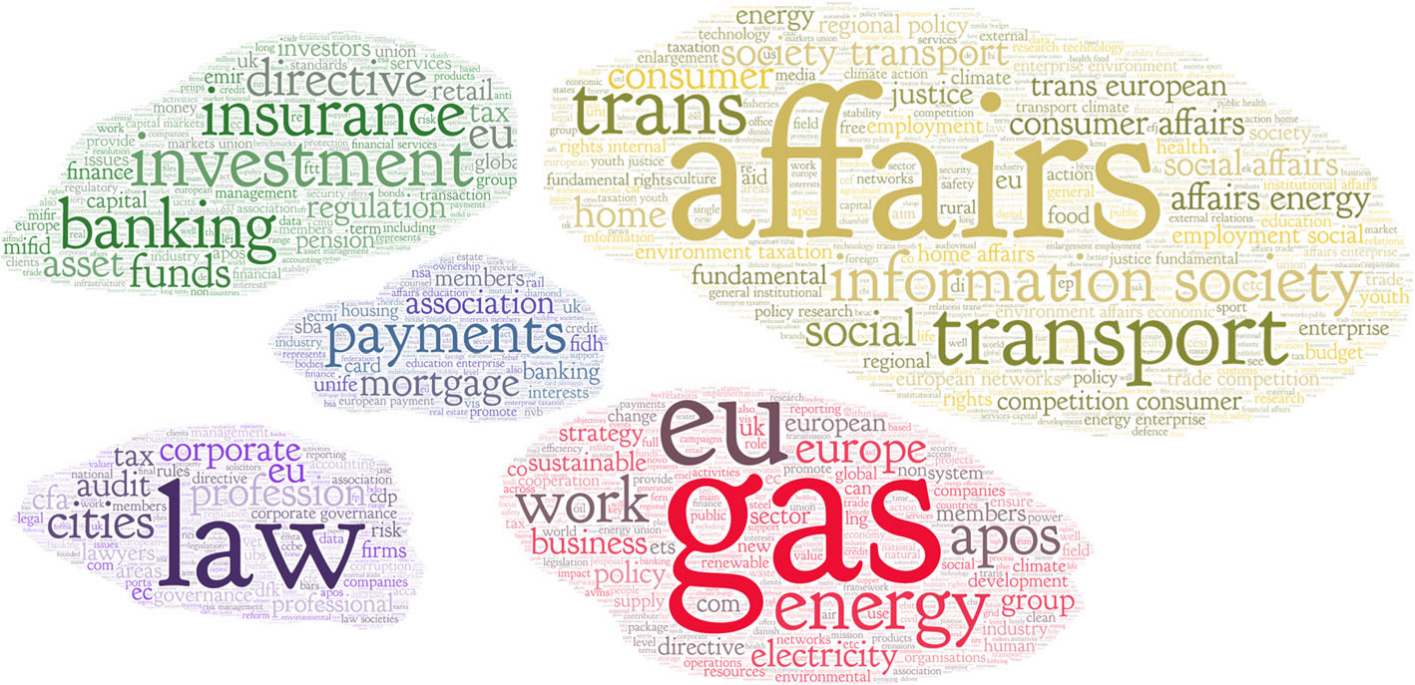
Tag clouds of the five clusters of organizations. The tag clouds are constructed from the self-described goals and activities of the 565 lobby organizations. Size of the clouds is proportional to the number of organizations in them

The clustering results for *K*=5 are shown in Table 7 and in Fig. 3. Table 7 shows, for each cluster, its short name, the number of organizations covered, and top ten centroid terms with their weights. Figure 3 shows the tag clouds, with the fifty most important centroid terms for each cluster, and size approximately proportional to the number of organizations.

**Table 7:** Results of clustering (*K*=5) applied to textual self-descriptions of organizations

Cluster	Org	Top centroid terms
Banking	140	Investment(0.176), banking(0.144), insurance(0.144), directive(0.137), regulation(0.129), funds(0.108), pension(0.106), investors(0.091), asset(0.09), capital(0.088)
Transport	227	Affairs(0.146), information society(0.112), transport(0.112), society transport(0.106), competition consumer(0.105), consumer affairs(0.103), employment social(0.103), trans european(0.102), trans(0.102), affairs energy(0.102)
Payments	31	Payments(0.163), mortgage(0.146), association(0.128), members(0.124), banking(0.118), education enterprise(0.103), housing(0.103), european payment(0.097), interests(0.097), industry(0.096)
Law	44	Law(0.184), profession(0.112), cities(0.11), corporate(0.098), audit(0.088), professional(0.084), lawyers(0.081), governance(0.079), corporate governance(0.079), management(0.078)
Energy	123	Gas(0.152), energy(0.151), eu(0.135), electricity(0.097), work(0.094), europe(0.084), business(0.084), apos(0.082), sustainable(0.08), policy(0.076)

Each cluster is identified by a short name, the number of organizations it covers, and the top ten centroid terms with their weights

**Fig. 4:**
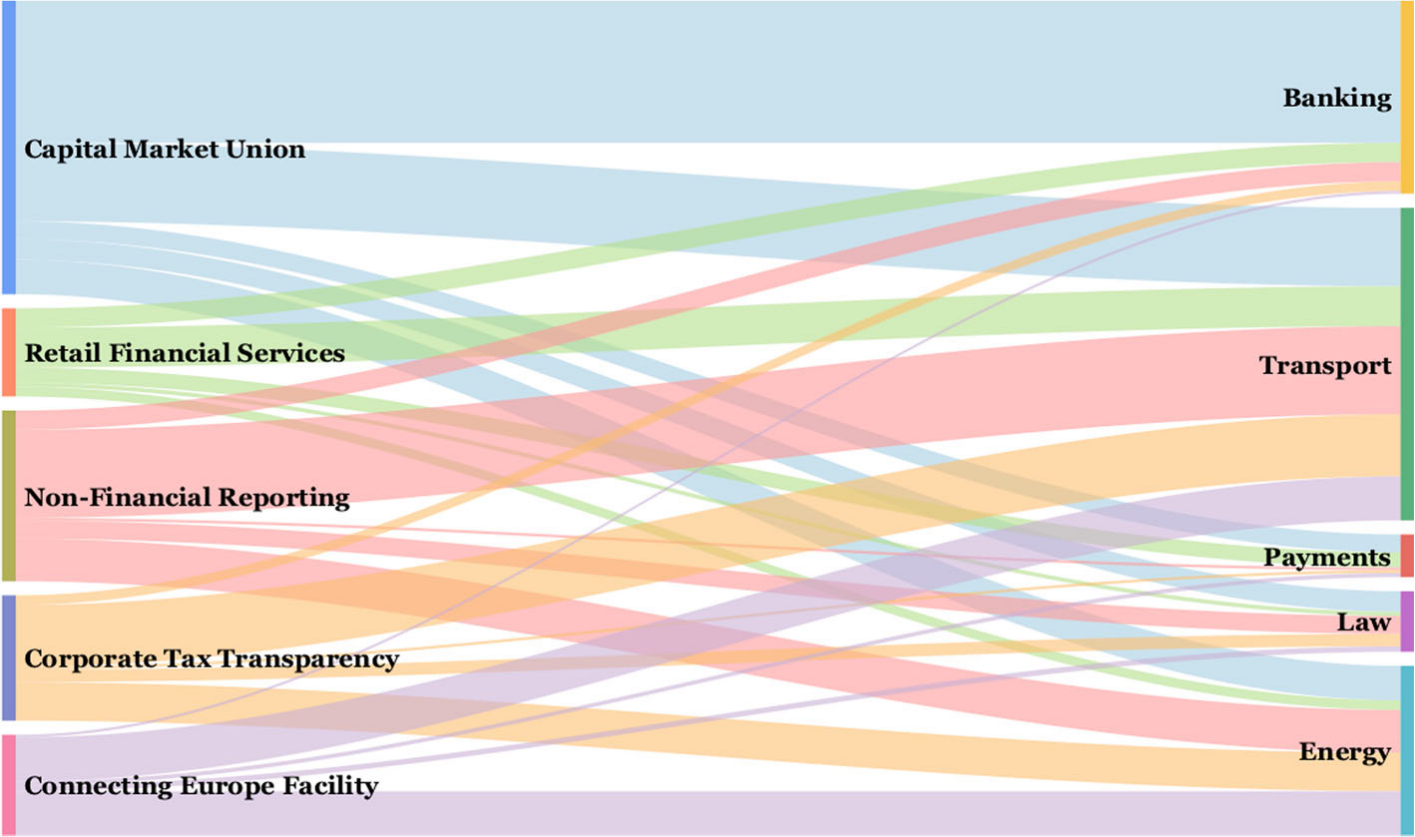
Relations between the topic communities and tag clouds. The Sankey diagram links the detected topic communities (left-hand side) to the clusters of the self-described goals and activities (right-hand side). We observe no significant correspondence between the topic communities and clusters, also confirmed by quantitative measures

The relation between the detected topic communities and the textual descriptions, encapsulated in the tag clouds, can be intuitively presented with a Sankey diagram. The diagram in Fig. 4 shows proportions and distribution of the 565 organizations in the topic communities and clusters of their descriptions. Thickness of links corresponds to the number of organizations that are present in both partitions.

The correspondence between both partitioning can be assessed by the *B*^3^ measure (Bagga and Baldwin 1998). *B*^3^ is considered the most appropriate measure for extrinsic evaluation of clustering (Amigó et al. 2009). It is similar to the Rand index, counting pairs of nodes in clusters, but is more sensitive in distinguishing small errors in big clusters from large number of small errors in small clusters. The *B*^3^ measure decomposes the evaluation into calculating the precision and recall associated with each node in two groupings. The correspondence between the two groupings is measured as the average value over all nodes, i.e., in our case all 565 organizations.

Let *N* be the set of all nodes in two groupings, say grouping 1 and 2. For each node *n*∈*N*, we denote with *L*(*n*) the set of nodes with the same group label as *n*, i.e., members of the same group (community or cluster, in our case) in grouping 1. With *C*(*n*), we denote the set of all nodes which are members of the same group as *n* in grouping 2. The *B*^3^ precision of a node *n*, *P*(*n*), is computed as the fraction of nodes which have the same label as *n* in both groupings, from all the nodes which are in the same group as *n* in grouping 2. Similarly, the *B*^3^ recall of a node *n*, *R*(*n*), is computed as the fraction of nodes with the same label in both groupings, from all the nodes with the same label as *n* in grouping 1. The precision and recall is then combined into the *F*_1_ score, a harmonic mean of the precision and recall:

$$ P(n) = \frac{\left|L(n)\cap C(n)\right|}{\left|C(n)\right|},\;\;\; \\ R(n) = \frac{\left|L(n)\cap C(n)\right|}{\left|L(n)\right|},\;\;\; \\ F_{1}(n) = 2\,\frac{P(n)\,R(n)}{P(n) + R(n)}. $$

The *F*_1_ score is a special case of Van Rijsbergen’s effectiveness measure (Van Rijsbergen 1979), where precision and recall can be combined with different weights. The precision, recall, and *F*_1_ score of a grouping is a micro average of the scores of all the nodes. The resulting scores between the detected topic communities and the clusters of descriptions are *P*=0.315 and *R*=0.342, yielding *F*_1_=0.328.

All the measures have relatively low values, and we can conclude that there is no significant matching, inclusion nor containment between the two groupings. This indicates that there might be considerable differences between the declared interests of the lobby organizations and their actual manifestation as captured by their answers to consultations. This result, which confirms our previous analysis (Sluban et al. 2017), is the main reason why in the current paper we focus on co-voting and profiling of the lobby organizations.

## Interactive exploration of the lobby profiles

**Fig. 5:**
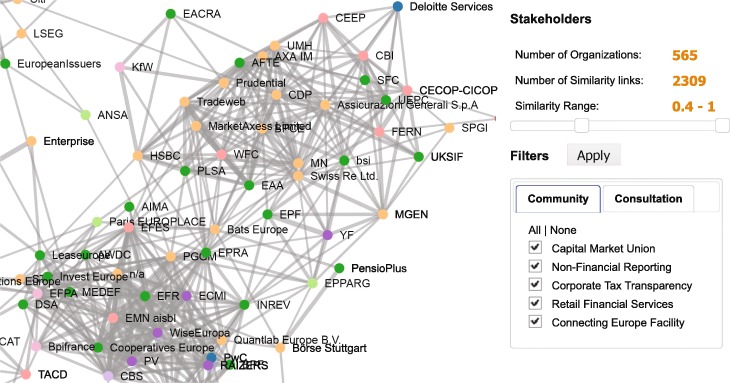
A screenshot of the *Lobby Profile Explorer*. On the right-hand size, the user can select consultation topics of interest for further explorations

We implemented the *Lobby Profile Explorer*, an interactive web application that supports exploration of the 565 lobby organizations. It presents a response network of the lobby organizations that responded to the 21 public consultations in the area of Banking and Finance. The implemented visualization has a variety of features, supporting in-depth exploration of the lobby network and pairwise comparison of the lobby profiles. A screenshot of the *Lobby Profile Explorer* interface is shown in Fig. 5. The web application and all the data are publicly accessible at https://simpolproject.eu/tools/lobby-profile-explorer/ and at https://kt.ijs.si/lobby/.

The response network is constructed from the responses to public consultations in terms of pairwise cosine similarities between the lobby organizations. The *Lobby Profile Explorer* supports selection of a range of similarity links to display in the network. Furthermore, the scope of the network, i.e., lobby organizations, can be refined by selecting specific consultations or individual topic communities with shared predominant interests, i.e., common consultations, as described in “Topic communities of responding organizations” subsection.

**Fig. 6:**
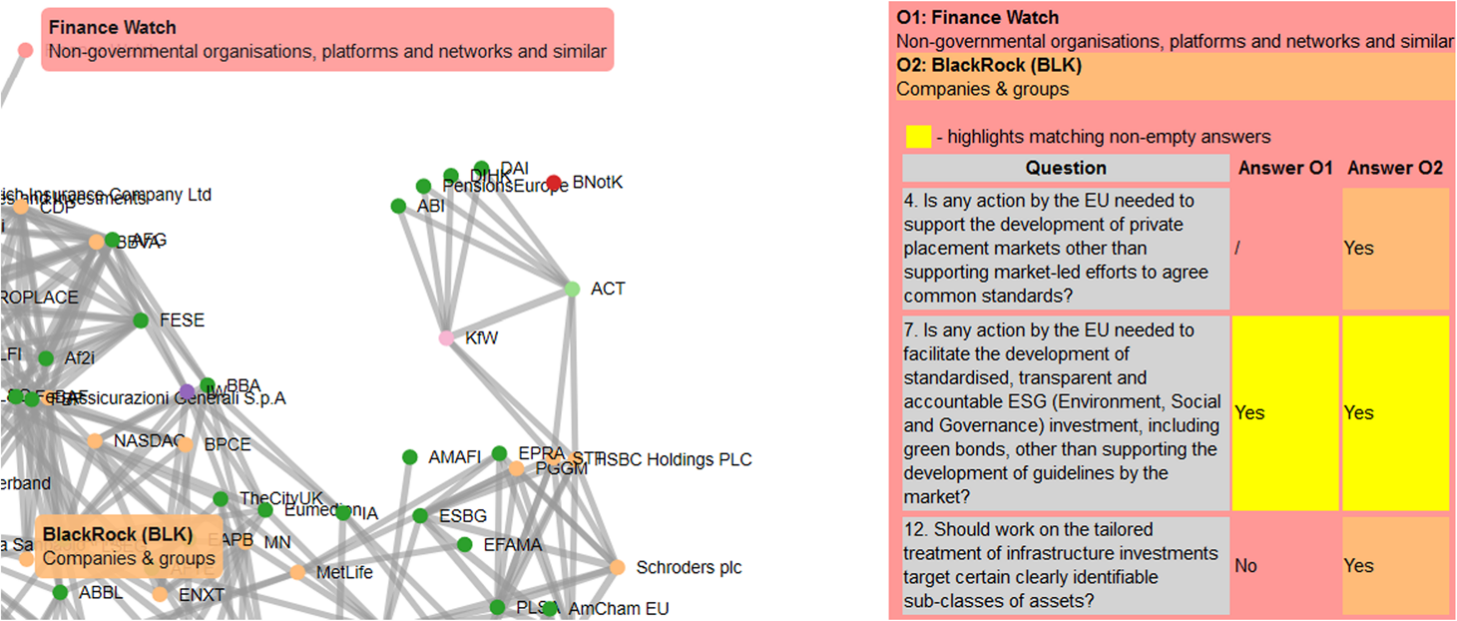
Interactive exploration and comparison of the lobby organizations. On the left-hand side is a network of organizations, linked by similar responses to the same consultations. On the right-hand side is a selected questionnaire, comparing answers by two selected lobby organizations

In addition to the zoom and pan features, the visualization allows to explore and compare specific lobby organization responses. By hovering over or clicking on a lobby node, a panel with the organization information and answers to specific questions is displayed. While the panel is open, a selection (click) of another lobby node in the network will show a comparison of the answers the two organizations provided and highlight the matches. Such an in-depth comparison of the responses of two lobby organizations (*Finance Watch* and *BlackRock*) to a selected consultation is illustrated in Fig. 6.

## Conclusions

We present how publicly accessible information can be used to assess the positions and leanings of major lobby organizations in the policy creation process. We focus on policy reforms in the area of Banking and Finance, and use data from the EU Transparency Register, and the EC public consultations. By combining methods from information retrieval, text mining, and network analysis we study different aspects of the lobby organizations which engage in policy shaping.

Our analysis shows that the categories representing the organization type do not align well with the clusters based on their declared goals and activities. Instead, responses to common consultations and similar answers to questions better characterize the true standings and leanings of the lobby organizations. From the organizations’ consultation responses we construct a response network representing inter-organization policy preferences. The community structure of this network reveals information about organizations’ activities and similarities that cannot be obtained from the organizations’ self-description of their goals and activities. This implies that the network analysis adds an important aspect that is complementary to text analysis in the understanding of how lobby organizations engage in the policy making process.

Our findings suggest that if we want to build a map of the policy making arena we should categorize lobby organizations based on their responses to policy issues via the consultations, rather than based on their general self-declared goals and activities, or based on their formal organization type categorization. Indeed, modeling the similarities of organizations’ positions in the consultations by means of networks enables not only to discover the community structure revealing actual common fields of engagement and interest, but offers also an intuitive representation of the lobbying ecosystem.

Building a consensus among stakeholders and a perception of transparency on stakeholders’ roles are crucial for a stable policy making process, as highlighted by the EU Better Regulation Agenda. However, as we show here, understanding stakeholders’ positions cannot simply rely on their static ex-ante categorizations. In contrast, it requires to take into account the actual positions of stakeholders, embedded in the context of the topic. Our work makes therefore a contribution to this issue by providing a new methodology to carry out such an analysis.

This work represents only the first step of a novel approach towards building maps of the policy arena. Future work will analyze how the design of the consultations could be improved in order to better identify the positions of the stakeholders with respect to the policy issues. The insights from this type of analysis and its future development can support the current EU policy agenda on increasing the transparency of the policy making process by enabling stakeholders and citizens to better understand which interests the various organizations represent and how they are influencing the policy debates.

## Abbreviations

*A**l**p**h**a*: Krippendorff’s Alpha agreement measure
EC: European commission
TR: Transparency register
JSD: Jensen-Shannon divergence
